# Mortuary Statistics

**Published:** 1880-01

**Authors:** 


MONTHLY RETURN

OF

### DEATHS AND INTERMENTS

### IN BALTIMORE.

| CAUSES OF DEATH. | ADULTS. | MINORS. | CAUSES OF DEATH. | ADULTS. | MINORS. |
|---|---|---|---|---|---|
| Abscess Lungs | 1 | | Dropsy, General | 6 | 3 |
| " Brain | | 1 | Disease of the Brain | 1 | |
| " Liver | 1 | | " " Heart | 26 | 2 |
| " Hepatic | 1 | | Drowned | 2 | |
| Aneurism of Aorta | | | Dysentery | 2 | |
| Anemia | | 1 | Empyemia | 1 | |
| Angina Pectoris | 1 | | Excessive Obesity | 1 | |
| Apoplexy | 8 | | Entero Colitis | | 1 |
| Asthenia | 1 | 3 | Endocarditis | 2 | 1 |
| Albumenuria | 1 | 2 | Erysipelas | 2 | 33 |
| Adynamia | | 1 | Fever, Malarial | | 1 |
| Abortion | 1 | | " Typhus | 1 | |
| Ascites | 4 | | " Puerperal | 1 | |
| Bright's Disease | 5 | 1 | " Remittent | | 2 |
| Burns | 2 | 6 | " Scarlet | | 47 |
| Capillary Bronchitis | | 7 | " Typhoid | 16 | 9 |
| Cancer, Diffused | 1 | | " Typho Malarial | 7 | |
| " Breast | 1 | | Fracture, Skull | 1 | |
| " Liver | 2 | | Gangrene of Lungs | 1 | |
| " Stomach | 9 | | Gastro Enteritis | 1 | 4 |
| " Uterus | 5 | | Hemorrhage, Brain | 1 | |
| " Pancreas | 1 | | Hernia, Strangulated | 1 | |
| Cerebral Embloism | | 1 | Hydrocephalus | | 1 |
| Child Birth | 2 | | Hepatic Colic | 1 | |
| Colitis | | | Inanition | 5 | 4 |
| Congestion of Brain | 3 | 5 | Inflammation of Bladder | 1 | |
| " Lungs | 2 | 3 | " Brain | 1 | 4 |
| Cholera Infantum | | 3 | " Bowels | | 2 |
| Casualties | 4 | | " Bronchi | 7 | 3 |
| Compression of Brain | | 1 | " Kidney | 2 | 1 |
| Consumption of Bowels | 1 | | " Larynx | | 4 |
| " Lungs | 87 | 21 | " Peritoneum | 1 | 1 |
| Convulsions | | 24 | " Pleura | 3 | |
| Convulsions, Puerperal | 2 | | " Stomach | 3 | 1 |
| Croup | | 43 | " Tonsils | | 10 |
| Congestive Chill | | 1 | Intestinal Catarrh | 1 | 3 |
| Dentition | | 7 | Intussusception | | 1 |
| Diarrhœa | 1 | 1 | Instrumental Labor | | 1 |
| Diphtheria | | 35 | Intemperance | 2 | |

| CAUSES OF DEATH. | ADULTS. | MINORS. | CAUSES OF DEATH. | ADULTS. | MINORS. |
|---|---|---|---|---|---|
| Jaundice | 1 | .... | Scrofula | 1 | 4 |
| Laryngitis | .... | .... | Softening of Brain | 3 | .... |
| Malignant Disease of Throat | 1 | .... | Septicæmia | 1 | .... |
| Mal Nutrition | .... | 1 | Stricture, Intestines | 1 | .... |
| Marasmus | .... | 15 | Suicide | 1 | .... |
| Meningitis | .... | 9 | " Opium | 1 | .... |
| " Tubercular | .... | 7 | Syphilis | .... | 2 |
| " Cerebro Spinal | .... | 1 | Tabes Mesenterica | .... | 1 |
| Malformation | .... | 1 | Tetanus | .... | 1 |
| Murder | 1 | .... | Trismus Nascentium | .... | 4 |
| Old Age | 28 | .... | Typhilitis | 1 | .... |
| Paralysis | 14 | .... | Tumor, Ovarian | 4 | .... |
| Puerperal Metritis | 1 | .... | Uremia | 2 | .... |
| Pyæmia | 1 | .... | Unknown Adult | 1 | .... |
| Pneumonia | 14 | 16 | " Infantile | .... | 4 |
| " Typhoid | 1 | 1 | Whooping Cough | .... | 4 |
| " Pleura | 1 | 1 | Wounds | 1 | 1 |
| Rheumatism, Heart | 1 | .... | | | |
| " Inflammatory | 2 | .... | Totals | 329 | 339 |

THE DEATHS REGISTERED IN THE MONTH INCLUDE

| | | | |
|---|---|---|---|
| Under 1 year | 110 | From 30 to 40 | 58 |
| From 1 to 2 | 80 | " 40 to 50 | 49 |
| " 2 to 5 | 58 | " 50 to 60 | 38 |
| " 5 to 10 | 75 | " 60 to 70 | 41 |
| " 10 to 15 | 13 | " 70 to 80 | 49 |
| " 15 to 20 | 22 | " 80 to 90 | 23 |
| " 20 to 30 | 61 | " 90 to 100 | 4 |

MALES, 322. FEMALES, 369. BOYS, 166. GIRLS, 194.

NATIVITY, ETC.

| | | | |
|---|---|---|---|
| United States (White) | 421 | From the Penitentiary | 1 |
| Foreign | 121 | " City Jail | ...... |
| Inquest Cases | 18 | " Hospitals | 28 |
| People of Color | 149 | | |

| | MALES. | FEMALES | TOTAL. |
|---|---|---|---|
| Premature Birth | 5 | 6 | 11 |
| Still Born | 37 | 27 | 64 |

